# Supplementary material for: Modeling glioblastoma heterogeneity as a dynamic network of cell states
Source: Mol Syst Biol. 2021 Sep 16;17(9):e10105. doi: 10.15252/msb.202010105 (PMC8444284; doi:10.15252/msb.202010105)
Supplement: Supplementary file 5 — Source Data for Figure 3 [file MSB-17-e10105-s001.zip › Figure3A_sourcedata/GSEA_3065/hallmarks_state4.GseaPreranked.1623416296068/HALLMARK_G2M_CHECKPOINT.html]

Details for gene set HALLMARK\_G2M\_CHECKPOINT[GSEA]

|  || Dataset | state4 |
| Phenotype | NoPhenotypeAvailable |
| Upregulated in class | na\_neg |
| GeneSet | HALLMARK\_G2M\_CHECKPOINT |
| Enrichment Score (ES) | -0.8060821 |
| Normalized Enrichment Score (NES) | -3.041087 |
| Nominal p-value | 0.0 |
| FDR q-value | 0.0 |
| FWER p-Value | 0.0 |
Table: GSEA Results Summary

  

Fig 1: Enrichment plot: HALLMARK\_G2M\_CHECKPOINT      
 Profile of the Running ES Score & Positions of GeneSet Members on the Rank Ordered List

  

| PROBE | GENE SYMBOL | GENE\_TITLE | RANK IN GENE LIST | RANK METRIC SCORE | RUNNING ES | CORE ENRICHMENT || 1 | MARCKS |  |  | 27 | 0.363 | 0.0056 | No |
| 2 | ATRX |  |  | 725 | 0.111 | -0.1053 | No |
| 3 | PURA |  |  | 1242 | 0.074 | -0.1877 | No |
| 4 | MT2A |  |  | 1542 | 0.060 | -0.2349 | No |
| 5 | CDKN1B |  |  | 1650 | 0.057 | -0.2508 | No |
| 6 | TLE3 |  |  | 1668 | 0.056 | -0.2520 | No |
| 7 | DMD |  |  | 1710 | 0.055 | -0.2572 | No |
| 8 | PAFAH1B1 |  |  | 1964 | 0.048 | -0.2973 | No |
| 9 | ARID4A |  |  | 2045 | 0.046 | -0.3091 | No |
| 10 | CBX1 |  |  | 2104 | 0.044 | -0.3174 | No |
| 11 | CUL5 |  |  | 2536 | 0.034 | -0.3869 | No |
| 12 | CDKN2C |  |  | 2822 | 0.028 | -0.4327 | No |
| 13 | RAD23B |  |  | 2951 | 0.026 | -0.4530 | No |
| 14 | HIRA |  |  | 3043 | 0.024 | -0.4672 | No |
| 15 | FOXN3 |  |  | 3161 | 0.022 | -0.4857 | No |
| 16 | AMD1 |  |  | 3213 | 0.021 | -0.4935 | No |
| 17 | ABL1 |  |  | 3254 | 0.020 | -0.4995 | No |
| 18 | TGFB1 |  |  | 3283 | 0.019 | -0.5035 | No |
| 19 | YTHDC1 |  |  | 3393 | 0.016 | -0.5209 | No |
| 20 | CDK4 |  |  | 3394 | 0.016 | -0.5205 | No |
| 21 | UPF1 |  |  | 3773 | 0.002 | -0.5823 | No |
| 22 | CHMP1A |  |  | 3804 | 0.000 | -0.5872 | No |
| 23 | RASAL2 |  |  | 3859 | -0.002 | -0.5959 | No |
| 24 | CUL3 |  |  | 3899 | -0.003 | -0.6022 | No |
| 25 | NOTCH2 |  |  | 3944 | -0.006 | -0.6092 | No |
| 26 | TNPO2 |  |  | 3976 | -0.008 | -0.6141 | No |
| 27 | MEIS1 |  |  | 4218 | -0.023 | -0.6529 | No |
| 28 | PRPF4B |  |  | 4282 | -0.027 | -0.6624 | No |
| 29 | GSPT1 |  |  | 4383 | -0.034 | -0.6778 | No |
| 30 | TFDP1 |  |  | 4389 | -0.035 | -0.6777 | No |
| 31 | EWSR1 |  |  | 4473 | -0.042 | -0.6901 | No |
| 32 | UCK2 |  |  | 4516 | -0.045 | -0.6957 | No |
| 33 | MYC |  |  | 4618 | -0.053 | -0.7108 | No |
| 34 | ODF2 |  |  | 4621 | -0.053 | -0.7096 | No |
| 35 | SYNCRIP |  |  | 4908 | -0.075 | -0.7543 | No |
| 36 | SMARCC1 |  |  | 4941 | -0.078 | -0.7574 | No |
| 37 | SLC7A1 |  |  | 4953 | -0.079 | -0.7570 | No |
| 38 | MEIS2 |  |  | 5012 | -0.084 | -0.7641 | No |
| 39 | KMT5A |  |  | 5031 | -0.085 | -0.7647 | No |
| 40 | HSPA8 |  |  | 5041 | -0.086 | -0.7638 | No |
| 41 | TOP1 |  |  | 5082 | -0.090 | -0.7679 | No |
| 42 | JPT1 |  |  | 5145 | -0.096 | -0.7753 | No |
| 43 | CDC25A |  |  | 5172 | -0.099 | -0.7769 | No |
| 44 | PLK4 |  |  | 5210 | -0.102 | -0.7801 | No |
| 45 | SRSF1 |  |  | 5281 | -0.108 | -0.7885 | No |
| 46 | RPA2 |  |  | 5372 | -0.115 | -0.8001 | No |
| 47 | SAP30 |  |  | 5390 | -0.117 | -0.7996 | No |
| 48 | EXO1 |  |  | 5410 | -0.120 | -0.7994 | No |
| 49 | DKC1 |  |  | 5452 | -0.125 | -0.8026 | Yes |
| 50 | KIF5B |  |  | 5464 | -0.127 | -0.8009 | Yes |
| 51 | RBL1 |  |  | 5472 | -0.128 | -0.7985 | Yes |
| 52 | SLC7A5 |  |  | 5488 | -0.131 | -0.7974 | Yes |
| 53 | RAD21 |  |  | 5496 | -0.131 | -0.7949 | Yes |
| 54 | NEK2 |  |  | 5505 | -0.132 | -0.7925 | Yes |
| 55 | KPNB1 |  |  | 5514 | -0.133 | -0.7901 | Yes |
| 56 | CCNF |  |  | 5562 | -0.137 | -0.7940 | Yes |
| 57 | BUB3 |  |  | 5583 | -0.141 | -0.7934 | Yes |
| 58 | HMGB3 |  |  | 5584 | -0.142 | -0.7895 | Yes |
| 59 | POLA2 |  |  | 5615 | -0.146 | -0.7903 | Yes |
| 60 | NUP50 |  |  | 5619 | -0.147 | -0.7868 | Yes |
| 61 | POLE |  |  | 5624 | -0.147 | -0.7833 | Yes |
| 62 | G3BP1 |  |  | 5627 | -0.148 | -0.7796 | Yes |
| 63 | MCM6 |  |  | 5644 | -0.151 | -0.7780 | Yes |
| 64 | CASP8AP2 |  |  | 5646 | -0.152 | -0.7739 | Yes |
| 65 | TRA2B |  |  | 5652 | -0.152 | -0.7705 | Yes |
| 66 | TTK |  |  | 5684 | -0.157 | -0.7712 | Yes |
| 67 | HNRNPD |  |  | 5705 | -0.161 | -0.7700 | Yes |
| 68 | ILF3 |  |  | 5722 | -0.165 | -0.7681 | Yes |
| 69 | PRIM2 |  |  | 5736 | -0.169 | -0.7655 | Yes |
| 70 | BARD1 |  |  | 5779 | -0.179 | -0.7674 | Yes |
| 71 | MCM2 |  |  | 5785 | -0.180 | -0.7633 | Yes |
| 72 | SLC12A2 |  |  | 5790 | -0.181 | -0.7589 | Yes |
| 73 | POLQ |  |  | 5818 | -0.187 | -0.7582 | Yes |
| 74 | PRC1 |  |  | 5835 | -0.192 | -0.7555 | Yes |
| 75 | PLK1 |  |  | 5836 | -0.192 | -0.7501 | Yes |
| 76 | NSD2 |  |  | 5887 | -0.203 | -0.7527 | Yes |
| 77 | BRCA2 |  |  | 5910 | -0.210 | -0.7505 | Yes |
| 78 | SNRPD1 |  |  | 5911 | -0.210 | -0.7447 | Yes |
| 79 | RACGAP1 |  |  | 5953 | -0.223 | -0.7452 | Yes |
| 80 | CENPA |  |  | 5960 | -0.226 | -0.7399 | Yes |
| 81 | XPO1 |  |  | 5964 | -0.227 | -0.7341 | Yes |
| 82 | SLC38A1 |  |  | 5972 | -0.228 | -0.7290 | Yes |
| 83 | NCL |  |  | 5974 | -0.228 | -0.7228 | Yes |
| 84 | TROAP |  |  | 5983 | -0.232 | -0.7177 | Yes |
| 85 | STMN1 |  |  | 5993 | -0.236 | -0.7126 | Yes |
| 86 | KIF2C |  |  | 5996 | -0.237 | -0.7064 | Yes |
| 87 | SRSF10 |  |  | 6004 | -0.239 | -0.7009 | Yes |
| 88 | KNL1 |  |  | 6007 | -0.240 | -0.6946 | Yes |
| 89 | HNRNPU |  |  | 6008 | -0.241 | -0.6879 | Yes |
| 90 | SMC1A |  |  | 6017 | -0.245 | -0.6824 | Yes |
| 91 | SFPQ |  |  | 6022 | -0.247 | -0.6762 | Yes |
| 92 | CHEK1 |  |  | 6025 | -0.248 | -0.6697 | Yes |
| 93 | AURKA |  |  | 6026 | -0.248 | -0.6628 | Yes |
| 94 | BUB1 |  |  | 6045 | -0.266 | -0.6584 | Yes |
| 95 | CDC45 |  |  | 6063 | -0.276 | -0.6535 | Yes |
| 96 | MCM5 |  |  | 6069 | -0.278 | -0.6466 | Yes |
| 97 | FBXO5 |  |  | 6084 | -0.287 | -0.6410 | Yes |
| 98 | KIF22 |  |  | 6089 | -0.290 | -0.6336 | Yes |
| 99 | CCNA2 |  |  | 6100 | -0.297 | -0.6270 | Yes |
| 100 | CDC6 |  |  | 6103 | -0.299 | -0.6190 | Yes |
| 101 | KIF11 |  |  | 6109 | -0.304 | -0.6114 | Yes |
| 102 | MCM3 |  |  | 6113 | -0.307 | -0.6034 | Yes |
| 103 | LBR |  |  | 6118 | -0.312 | -0.5954 | Yes |
| 104 | SQLE |  |  | 6123 | -0.316 | -0.5873 | Yes |
| 105 | SRSF2 |  |  | 6128 | -0.321 | -0.5791 | Yes |
| 106 | HMGA1 |  |  | 6132 | -0.324 | -0.5706 | Yes |
| 107 | LMNB1 |  |  | 6134 | -0.325 | -0.5618 | Yes |
| 108 | DDX39A |  |  | 6137 | -0.328 | -0.5530 | Yes |
| 109 | TACC3 |  |  | 6138 | -0.328 | -0.5439 | Yes |
| 110 | CCNB2 |  |  | 6140 | -0.329 | -0.5349 | Yes |
| 111 | NDC80 |  |  | 6142 | -0.330 | -0.5260 | Yes |
| 112 | AURKB |  |  | 6143 | -0.332 | -0.5168 | Yes |
| 113 | HMMR |  |  | 6148 | -0.340 | -0.5080 | Yes |
| 114 | E2F1 |  |  | 6152 | -0.343 | -0.4990 | Yes |
| 115 | CDC20 |  |  | 6154 | -0.344 | -0.4896 | Yes |
| 116 | KIF15 |  |  | 6158 | -0.346 | -0.4805 | Yes |
| 117 | EZH2 |  |  | 6159 | -0.348 | -0.4709 | Yes |
| 118 | CDKN3 |  |  | 6160 | -0.349 | -0.4612 | Yes |
| 119 | KIF23 |  |  | 6162 | -0.351 | -0.4516 | Yes |
| 120 | HMGN2 |  |  | 6163 | -0.351 | -0.4419 | Yes |
| 121 | KIF4A |  |  | 6164 | -0.355 | -0.4321 | Yes |
| 122 | KIF20B |  |  | 6172 | -0.373 | -0.4229 | Yes |
| 123 | ODC1 |  |  | 6175 | -0.377 | -0.4128 | Yes |
| 124 | DTYMK |  |  | 6177 | -0.381 | -0.4024 | Yes |
| 125 | DBF4 |  |  | 6180 | -0.387 | -0.3920 | Yes |
| 126 | CHAF1A |  |  | 6183 | -0.396 | -0.3813 | Yes |
| 127 | NASP |  |  | 6187 | -0.403 | -0.3706 | Yes |
| 128 | MYBL2 |  |  | 6190 | -0.408 | -0.3596 | Yes |
| 129 | GINS2 |  |  | 6194 | -0.420 | -0.3485 | Yes |
| 130 | CCND1 |  |  | 6203 | -0.454 | -0.3373 | Yes |
| 131 | PBK |  |  | 6207 | -0.465 | -0.3249 | Yes |
| 132 | BIRC5 |  |  | 6214 | -0.484 | -0.3124 | Yes |
| 133 | SMC4 |  |  | 6219 | -0.490 | -0.2995 | Yes |
| 134 | NUSAP1 |  |  | 6223 | -0.507 | -0.2860 | Yes |
| 135 | CENPE |  |  | 6226 | -0.511 | -0.2721 | Yes |
| 136 | SMC2 |  |  | 6229 | -0.518 | -0.2581 | Yes |
| 137 | TMPO |  |  | 6233 | -0.522 | -0.2441 | Yes |
| 138 | CKS1B |  |  | 6234 | -0.523 | -0.2296 | Yes |
| 139 | KPNA2 |  |  | 6236 | -0.543 | -0.2147 | Yes |
| 140 | ORC6 |  |  | 6238 | -0.585 | -0.1987 | Yes |
| 141 | CKS2 |  |  | 6239 | -0.585 | -0.1825 | Yes |
| 142 | UBE2S |  |  | 6244 | -0.617 | -0.1660 | Yes |
| 143 | PTTG1 |  |  | 6245 | -0.622 | -0.1488 | Yes |
| 144 | CDK1 |  |  | 6246 | -0.627 | -0.1314 | Yes |
| 145 | MAD2L1 |  |  | 6247 | -0.628 | -0.1140 | Yes |
| 146 | TPX2 |  |  | 6253 | -0.667 | -0.0964 | Yes |
| 147 | UBE2C |  |  | 6255 | -0.750 | -0.0758 | Yes |
| 148 | TOP2A |  |  | 6260 | -0.814 | -0.0539 | Yes |
| 149 | MKI67 |  |  | 6261 | -0.882 | -0.0294 | Yes |
| 150 | CENPF |  |  | 6263 | -1.068 | -0.0000 | Yes |
Table: GSEA details [plain text format]

  

Fig 2: HALLMARK\_G2M\_CHECKPOINT: Random ES distribution      
 Gene set null distribution of ES for **HALLMARK\_G2M\_CHECKPOINT**
